# Supplementary material for: Plasmonic gain in current biased tilted Dirac nodes
Source: Nat Commun. 2022 Dec 12;13:7667. doi: 10.1038/s41467-022-35139-y (PMC9744885; doi:10.1038/s41467-022-35139-y)
Supplement: Supplementary file 1 — Supplementary Information [file 41467_2022_35139_MOESM1_ESM.pdf]

Plasmonic gain in current biased tilted Dirac nodes: Supplementary Information

Sang Hyun Park,[1] Michael Sammon,[1] Eugene Mele,[2] and Tony Low[1,*]

[1]*Department of Electrical & Computer Engineering, University of Minnesota, Minneapolis, Minnesota, 55455, USA*

[2]*Department of Physics and Astronomy, University of Pennsylvania, Philadelphia, Pennsylvania, 19104, USA*

Supplementary Note 1. Hydrodynamic model

To gain a better understanding of the plasmon modes with both electron and hole pockets, we use the classical hydrodynamic model introduced by[1]. Consider two charged fluids described by a background charge density n_{a0} and fluctuation $n_{a1}(\mathbf{r}, t)$ where $a = 1, 2$ label each fluid. The Euler equation of motion for the interacting electron and hole fluids is given as:

$$M_a \frac{\partial j_a}{\partial t} = -n_{a0} \nabla \int dr' \frac{e^2}{|\mathbf{r} - \mathbf{r}'|} \left[n_{11}(\mathbf{r}', t) + n_{21}(\mathbf{r}', t) \right]$$

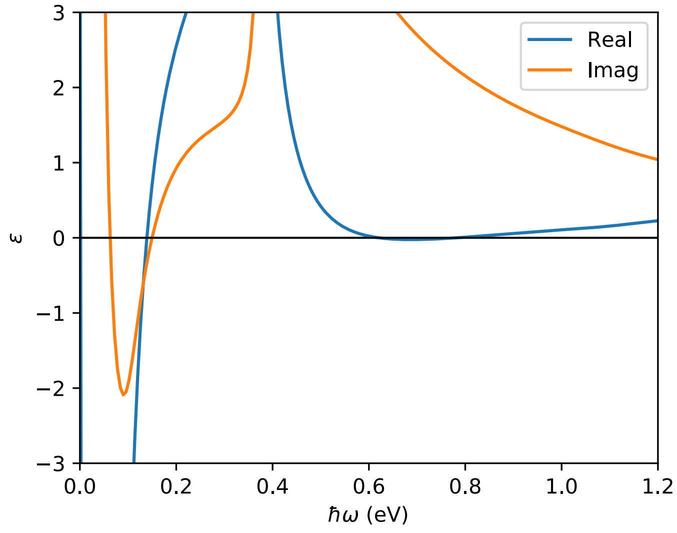

Supplementary Figure 1. **Real and imaginary part of $\epsilon(\omega)$ showing the plasmon solutions.**

* tlow@umn.edu

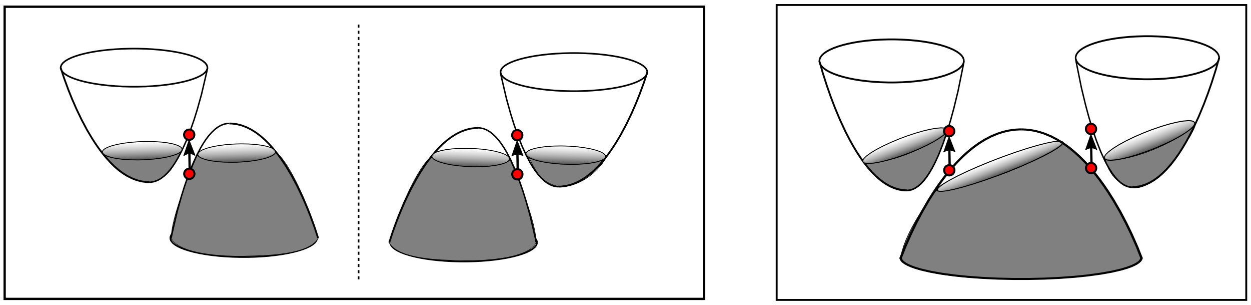

Supplementary Figure 2. Contributions to polarizability calculation for (left) tilted Dirac model and (right) WTe_2 band structure.

where M_a is the effective mass tensor. Using the continuity equation and Fourier transform we arrive at

$$\begin{bmatrix} \omega^2 - n_{10}V_q^{11}\left(\frac{q_x^2}{m_{1x}}+\frac{q_y^2}{m_{1y}}\right) & -n_{10}V_q^{12}\left(\frac{q_x^2}{m_{1x}}+\frac{q_y^2}{m_{1y}}\right) \\ -n_{20}V_q^{21}\left(\frac{q_x^2}{m_{2x}}+\frac{q_y^2}{m_{2y}}\right) & \omega^2 - n_{20}V_q^{22}\left(\frac{q_x^2}{m_{2x}}+\frac{q_y^2}{m_{2y}}\right) \end{bmatrix} \begin{bmatrix} n_{11}(\mathbf{q},\omega) \\ n_{21}(\mathbf{q},\omega) \end{bmatrix} = 0$$

where $V_q^{11} = V_q^{22} = V_q = e^2/2\epsilon_0 q$, $V_q^{12} = V_q^{21} = -V_{|q+q_{eh}|}$, and q_{eh} is the separation between the electron and hole pockets. For a type-II node with $E_F = 0\text{eV}$ we may assume that $n_{10} = n_{20} = n_0$ and $m_{1x} = m_{2x} = m_x$. Then the dispersion is found to be

$$\omega_\pm^2 = \frac{n_0 q_x^2}{m_x}\left(V_q \pm V_{|q+q_{\mathrm{eh}}|}\right)$$

which gives two split branches. The ω_+ solution corresponds to an out-of-phase oscillation of the electron and hole pockets while the ω_- solution is an in-phase oscillation. For $q = -0.06$ 1/Å, the real and imaginary part of the dielectric function are shown in supplementary fig. 1. Parameters of the Dirac model are identical to those used in fig. 2 of the main text. We see a lower energy solution with $\mathrm{Im}(\epsilon) < 0$ signifying a plasmon with gain. At higher energy we find a second solution with loss in agreement with the hydrodynamic theory analysis. This mode exists at energies much higher than the gain window and is not analyzed in detail for this work.

Supplementary Note 2. Double counting of valence band

When comparing the band structure of WTe_2 and the tilted Dirac model, it may seem as though we are counting the valence band twice by using two copies of the Dirac model. However, when calculating the polarizability, it is more important to capture the correct transitions rather than number of bands. For interband transitions, consider a direct transition ($q = 0$) at a given energy $\hbar\omega$. The transition as described by using two copies of the tilted Dirac model and by using the

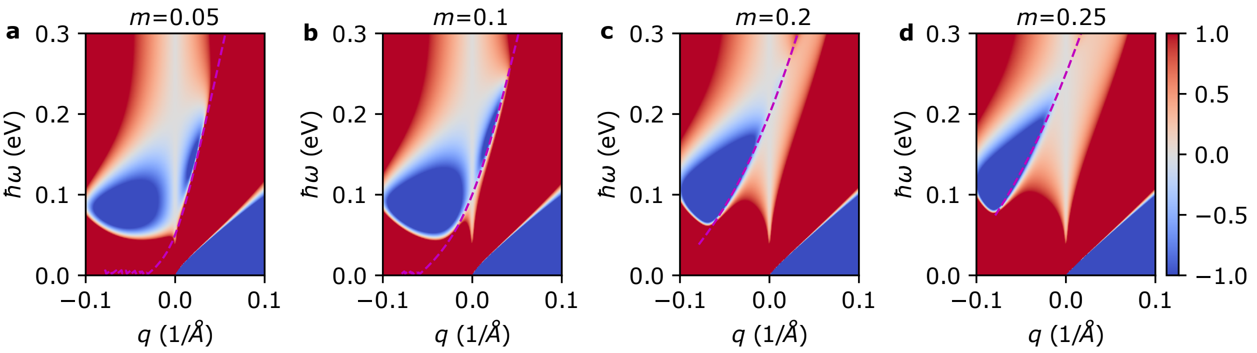

Supplementary Figure 3. **Change of the gain window as a function of mass parameter in Dirac model.**

bandstructure of WTe_2 are compared in supplementary fig. 2. It is easy to see that while the Dirac model includes two hole pockets, the number of direct transitions it accounts for is identical to the WTe_2 band structure. For intraband transitions in the valence band, note that the valence band given by the tilted Dirac model is asymmetric. Therefore an allowed transition at (q, ω) in the $\eta = +1$ node does not have a corresponding transition in the $\eta = -1$ node. Rather, since the band structures of the two nodes are mirror images of each other, for every transition (q, ω) in the $\eta = +1$ node, there will be a transition at $(-q, \omega)$ in the $\eta = -1$ node. This is exactly the required behavior for intraband transitions in the valence band for WTe_2.

Supplementary Note 3. Effect of electron hole pocket separation

In the main text, we have discussed that the gain window overlapping with the plasmon dispersion can be attributed to interband transitions. Hence the separation between electron and hole pockets must be an important factor in determining the size and position of the gain window. In terms of the Dirac model, the electron hole pocket separation is most significantly influenced by the mass term m. In supplementary fig. 3 we show the imaginary part of the dielectric function as m is varied. From this calculation we find that the gain window is indeed shifted. Applying strain to the material can be one way to tune the electron-hole pocket separation and thus shift the gain window.

Supplementary Note 4. Linear phase boundary

Here we derive an analytic relation between α and u_{th}. Recall that the energy dispersion relation of the tilted Dirac model is $E_{\pm} = tk \pm \left[v^2k^2 + (m/2 - \alpha k^2)^2\right]^{1/2}$. For $E_F = 0$ and $u = 0$ the Fermi

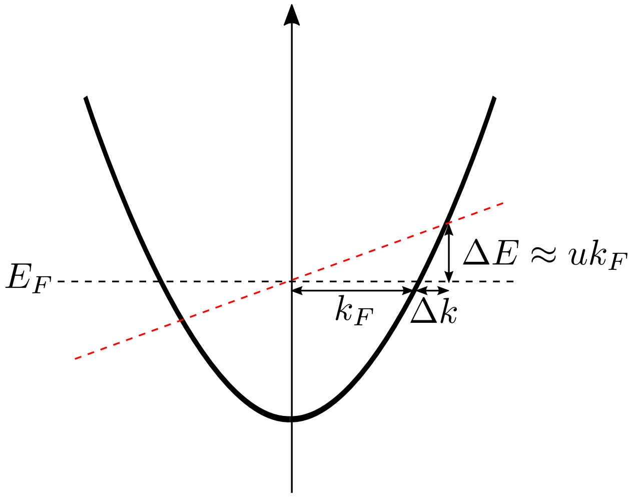

Supplementary Figure 4. **Schematic representation of the Fermi surface shift under the modified carrier distribution.**

wave vector is given by

$$k_F^2 = \frac{1}{2\alpha^2}\left(m\alpha + t^2 - v^2 \pm \sqrt{(v^2 - t^2 - m\alpha)^2 - m^2\alpha^2}\right). \tag{S1}$$

Then for $m = 0$ we simply find that $k_F = \sqrt{t^2 - v^2}/\alpha$. Now assume that the Fermi surface shift required for gain is independent of the parameters u and α since both parameters do not affect the separation of the electron and hole pockets. Taking the threshold Fermi surface shift to be Δk_{th} we may write

$$\Delta k_{th} = \frac{u_{th} k_F}{t + v} = \sqrt{\frac{t - v}{t + v}}\frac{u_{th}}{\alpha} \tag{S2}$$

and therefore we find that $u_{th}/\alpha = C(v, t, m)$ where C is a constant dependent on the parameters v, t, m.

Supplementary Note 5. Threshold current density

In the manuscript our calculations and analysis is based on the parameter u which modifies the electron distribution to $f_u(E_{\mathbf{k}}) = \left(e^{(E_{\mathbf{k}} - \mathbf{u}\cdot\mathbf{k} - E_F)/k_B T} + 1\right)^{-1}$. Here we try to find the physical current density $\mathbf{J}_d$ from this distribution. In general, the current density is given by

$$\mathbf{J}_d = \frac{q}{L^2}\sum_{\mathbf{k}} \mathbf{v}(\mathbf{k}) n(\mathbf{k})$$

where $\mathbf{v}(\mathbf{k}) = \nabla_{\mathbf{k}} E/\hbar$, q is the carrier charge, and $n(\mathbf{k})$ is the carrier distribution function. Since our system has both electrons and holes, let us treat each case separately.

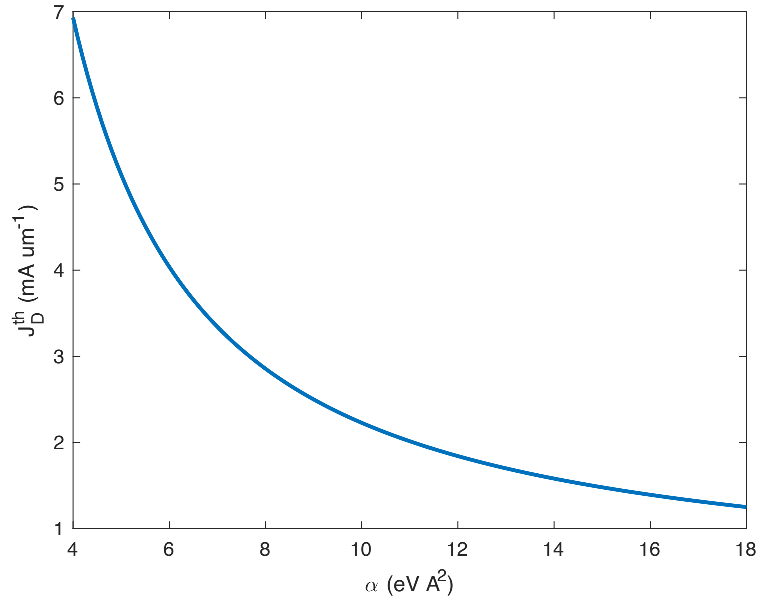

Supplementary Figure 5. **Threshold current density to achieve gain as a function of parameter α.**

For electrons, the energy dispersion is $E^{(\eta)}(\mathbf{k}) = t\eta k_x + [v^2k^2 + (m/2 - \alpha k^2)^2]^{1/2}$ where η is the node index. It is then straightforward to calculate the velocity,

$$v_{x,e}^{(\eta)} = \frac{1}{\hbar}\left[\eta t + k_x \frac{v - 2\alpha(m/2 - \alpha k^2)}{(v^2k^2 + (m/2 - \alpha k^2)^2)^{1/2}}\right].$$

We also know that the electrons pockets for the $\eta = \pm 1$ nodes at $u = 0$ are related by $n_e^{(\eta=+1)}(k_x, k_y) = n_e^{(\eta=-1)}(-k_x, k_y)$. Therefore, the total contribution to the current density from each of the nodes cancel out for zero drift velocity as expected, i.e. $\mathbf{j}_e^{(\eta=+1)} = -\mathbf{j}_e^{(\eta=-1)}$.

The dispersion of the band with the hole pocket is $E^{(\eta)}(\mathbf{k}) = t\eta k_x - [v^2k^2 + (m/2 - \alpha k^2)^2]^{1/2}$. Note that the energy and momentum here still refer to those of the electrons. To calculate the hole velocity we write

$$\mathbf{v}_h = \frac{1}{\hbar}\nabla_{\mathbf{k}_h} E_h(\mathbf{k}_h) = \frac{1}{\hbar}(-\nabla_{\mathbf{k}})(-E(-\mathbf{k})) = -\frac{1}{\hbar}\nabla_{\mathbf{k}} E(\mathbf{k}).$$

Since $q_e = -q_h$ and $n_e^{(\eta)}(k_x, k_y) = n_h^{(\eta)}(-k_x, k_y)$ we find that $\mathbf{j}_e^{(\eta)} = \mathbf{j}_h^{(\eta)}$. The electron and hole pockets of a given node have equal contributions to the total current density. Of course for zero drift, contributions from each node are opposite and thus cancel out giving zero current density. For $u \neq 0$, the contributions from each node no longer cancel and we have a non-zero current density.

With these results, we can plot the threshold current density as a function of the band parameter α. From the phase diagram in fig. 3 of the main text we found that the threshold for gain follows

the linear boundary $\alpha = 50.99u/v - 6.508$. Then using the results above we find the results shown in supplementary fig. 5 for the threshold current density.

[1] K. Sadhukhan, A. Politano, and A. Agarwal, Phys. Rev. Lett. **124**, 46803 (2020), arXiv:1904.10137.
